# Relationship between Human Evolution and Neurally Mediated Syncope Disclosed by the Polymorphic Sites of the Adrenergic Receptor Gene α2B-AR

**DOI:** 10.1371/journal.pone.0120788

**Published:** 2015-04-10

**Authors:** Tomoyoshi Komiyama, Takatsugu Hirokawa, Kyoko Sato, Akira Oka, Hiroshi Kamiguchi, Eiichiro Nagata, Hiroshi Sakura, Kuniaki Otsuka, Hiroyuki Kobayashi

**Affiliations:** 1 Department of Clinical Pharmacology, Tokai University School of Medicine, 143 Shimokasuya, Isehara, Kanagawa, 259–1193, Japan; 2 The National Institute of Advanced Industrial Science and Technology (AIST), Tokyo Waterfront Bio-IT Research Building 2-4-7 Aomi, Koto-ku, Tokyo, 135–0064, Japan; 3 Tokyo Women’s Medical University Medical Center East, 2-1-10 Higashiogu, Arakawa-ku, Tokyo, 116–8567, Japan; 4 The Institute of Medical Science, Tokai University, 143 Shimokasuya, Isehara, Kanagawa, 259–1193, Japan; 5 Support Center for Medical Research and Education, Tokai University, 143 Shimokasuya, Isehara, Kanagawa, 259–1193, Japan; 6 Department of Neurology, Tokai University School of Medicine, 143 Shimokasuya, Isehara, Kanagawa, 259–1193, Japan; Tokai University, JAPAN

## Abstract

The objective of this study was to clarify the effects of disease on neurally mediated syncope (NMS) during an acute stress reaction. We analyzed the mechanism of the molecular interaction and the polymorphisms of the alpha-2 adrenoreceptor (α2B-AR) gene as the potential psychiatric cause of incentive stress. We focused on the following three genotypes of the repeat polymorphism site at Glu 301–303 in the α2B-AR gene: Glu12/12, Glu12/9, and Glu9/9. On the basis of our clinical research, NMS is likely to occur in people with the Glu12/9 heterotype. To verify this, we assessed this relationship with the interaction of Gi protein and adenylate cyclase by *in silico* analysis of the Glu12/9 heterotype. By measuring the difference in the dissociation time of the Gi-α subunit twice, we found that the Glu12/9 heterotype suppressed the action of adenylate cyclase longer than the Glu homotypes. As this difference in the Glu repeat number effect is thought to be one of the causes of NMS, we investigated the evolutionary significance of the Glu repeat number. Glu8 was originally repeated in simians, while the Glu12 repeats occurred over time during the evolution of bipedalism in humans. Taken with the Glu12 numbers, NMS would likely become a defensive measure to prevent significant blood flow to the human brain.

## Introduction

The long-term goal of our research is to clarify the effects of disease on neurally mediated syncope (NMS) during an acute stress reaction. Despite the problems that frequent NMS poses to activities of daily living, its mechanism has not been elucidated. In addition, treatment is limited by past pathophysiology.

In the general population, syncope is a relatively frequent disease, with approximately 30% of individuals experiencing it at least once in their lifetime. Among such individuals, many have NMS. In recent years, approximately 20% of syncope cases were considered to be NMS [1, 2]. The most common explanation for NMS is that marked blood pooling in the capacitance vessels of the lower body when in an upright position causes a decrease in venous return, stroke volume, and cardiac output. The resultant decrease in blood pressure leads to an increase in sympathetic tone. This increase is thought to trigger a reflex loss of sympathetic tone and associated vagotonia, which lead to hypotension and/or bradycardia [3]. However, our findings from previous clinical research did not support this hypothesis [4].

We started by investigating the epidemiology of NMS during an acute stress reaction and analyzed the genetic polymorphisms of the adrenergic receptors from the perspective of psychiatric causes of incentive stress. Our research was the first to clarify the probable cause of syncope based on molecular biological analysis of the alpha-2 adrenoreceptor (α2B-AR) gene. The adrenergic receptor is deeply involved in the regulation of neurotransmitter release from adrenergic neurons in both the sympathetic and central nervous systems [3]. Moreover, polymorphisms of adrenergic receptor genes are known to be associated with the pathology and hemodynamics of heart failure in humans. Some studies have reported an association between diseases such as heart failure, diabetes, obesity, and polycystic ovary syndrome with these polymorphisms in European, African, and South American populations [5–10]. However, no studies have reported a relationship between NMS and polymorphisms of adrenergic receptor genes. Therefore, in the present study, we sought to clarify the relationship between the interaction of guanine nucleotide-binding protein i (Gi) protein and adenylate cyclase (AC) by *in silico* analysis. AC mediates the effects of Gi protein on the contraction and relaxation of blood vessels by acting on adrenergic receptor α1 or β2 [11–13]. For example, when the adrenergic subtype β2 receptor is activated, it promotes the binding of AC to Gs protein to create cyclic adenosine monophosphate (cAMP) [14] (S1 Fig). When cAMP activates protein kinase A, calcium ion channels are opened, which accelerates calcium uptake by the sarcoplasmic reticulum [15]. Therefore, the increase in calcium concentration is related to an increase in the contractile force of the smooth muscle [16].

Conversely, we believe that NMS is strongly related to the acquisition of upright bipedal walking in the course of human evolution. Modern apes and humans, which are considered to share a common evolutionary lineage, are differentiated by whether or not they are bipedal. It is thought that the evolution of bipedalism in humans led to various adaptive advantages, such as increased brain volume, the freedom to use both hands, and better long-distance vision. These advantages likely accelerated human evolution. However, bipedalism in humans has also led to various problems due to the effects of gravity on an upright posture [17–19], for example, low back pain, gastroptosia, hernia, and hemorrhoids. Therefore, we focus on syncope from the perspective that no appropriate method of treatment has yet been found.

In this study, we sought to clarify the relationship between α2B-AR gene repeat polymorphisms and clinical data on fainting by molecular analysis of the α2B-AR gene and Gi protein in NMS. We also continue the discussion on NMS in light of bipedalism and include some evolutionary events resulting from DNA homology and molecular evolution in humans and other primates.

## Results

### Clinical analysis of NMS subjects: Role of adrenaline and noradrenaline in NMS

We examined the concentrations of adrenaline (Ad) and noradrenaline (NA) during blood fluctuations in the head-up tilt (HUT) test (S2 Fig). To assess this, we recruited 9 subjects (5 men and 4 women) with NMS who were referred to our outpatient department and 11 healthy subjects (7 men and 4 women) who had never experienced syncope to investigate the epidemiology of the disease at the time of an acute stress reaction. First, we examined the concentration of Ad before and after the HUT test (S3a Fig). From the data shown in S3 Fig, all subject showed high concentrations of Ad after the HUT test; however, we did not find a significant difference between the groups. After the HUT test, the plasma NA concentration of the healthy subjects was 242.4 ± 87.6 pg/ml, and the concentration of the NMS patients was 344.6 ± 159.6 pg/ml. The NMS patients showed high Ad and NA concentrations before and after the HUT test (S3b Fig).

We then examined Ad and NA concentrations among the three different genotypes in humans (4 subjects with Glu12/12, 13 with Glu12/9, and 3 with Glu9/9). The NMS and healthy subjects with Glu12 (12/12 and 12/9) repeats had higher baseline Ad and NA concentrations than those with the Glu9 (9/9) repeats (S3c and S3d Fig). After the HUT test, the baseline Ad concentration did not change much in the three groups (S3c Fig). Conversely, our data showed that subjects with Glu12 repeats had higher NA concentrations than those with Glu9 repeats (S3d Fig). Our results might show that individuals who have Glu12 repeats could be more vulnerable to orthostatic stress than those with Glu9 repeats. All 9 NMS subjects had Glu12 repeats and did not have the Glu9/9 genotype. Our study suggested that individuals with the Glu12 repeats polymorphism of the α2B-AR gene may have the potential to develop NMS.

### Frequency of the α2B-AR: p.Glu 301–303 polymorphism in a Japanese population

As we confirmed the low frequency of Glu9 repeats, we next analyzed the frequency of Glu9 repeats among 281 healthy individuals of Japanese descent (Tables 1 and 2). Allele frequencies were calculated by allele counting. Analyses for possible deviations of the genotype distribution from that predicted for a population by the Hardy-Weinberg equilibrium were performed using Pearson’s chi-squared test. The three genotypes were found at the following frequency: type 12/12, 109; type 12/9, 127; and type 9/9, 45 (Table 1).

**Table 1:** Allele frequency of Glu9/9, 9/12, and 12/12 in 281 Japanese subjects.

Genotype	n	Observed frequency	Expected frequency from Hardy-Weinberg
Glu9/9	45	0.160	**0.149**
Glu9/12	127	0.452	**0.474**
Glu12/12	109	0.388	**0.377**
Total	281	1.000	1.000

**Table 2:** Allele frequency of Glu9 and Glu12 in 281 Japanese subjects.

Genotype	2n	Frequency
Glu9	217	**0.386**
Glu12	345	0.614
Total	562	1.000

A polymorphism of three glutamate amino acids was found at position 301–303. The allele frequency was 0.386 for Glu9 and 0.614 for Glu12 (Table 2). The genotype frequency was 0.149 for Glu9/9, 0.474 for Glu12/9, and 0.377 for Glu12/12 (Table 1). Next, we compared the Glu9 frequency using the data of Small et al. [20]. The Glu9 frequency was higher in Japanese individuals than in Caucasians and African-Americans, suggesting that the Glu12/9 heterotype is more common in Caucasians than in African-Americans. Moreover, the deviation between Glu9 and Glu12 was 0.605 (p < 0.05).

The null hypothesis is that the population is in Hardy-Weinberg equilibrium, while the alternative hypothesis is that the total ethnic population is not in equilibrium. There is one degree of freedom, and the 5% significance level for one degree of freedom is 3.84. Since the χ^2^ value was <3.84, the null hypothesis was accepted. In addition, the higher gene frequency of Glu12 (1 − 0.12 = 0.88) in Africans, as the origin of modern humans, suggests that the Glu12 frequency was higher in our ancestors (S1 Table).

**Fig 1:**
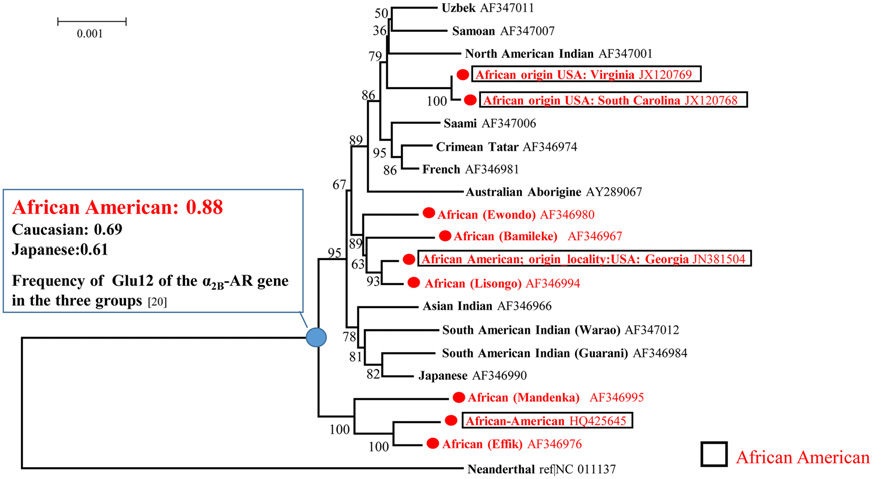
Phylogenetic tree constructed by the neighbor-joining method showing the complete mitochondrial DNA (mtDNA) region (16,750 bp) of *Homo sapiens sapiens* and *Homo sapiens neanderthalensis* and suggesting the evolution of the Glu12 repeats. On the basis of the differences in Glu12 frequency in the three groups, it is likely that most ancestors of modern-day Africans primarily had Glu12 repeats of the α2B-AR gene, and not Glu9 repeats.

Ingman et al. [21] reported that modern humans originated in Africa based on the results of complete mitochondrial DNA (mtDNA) analysis. Accordingly, we obtained samples, based on Ingman’s sequence data for complete mtDNA genomes, from GenBank to study the origin of the Glu repeats. The mtDNA genome data included 4 samples from African-Americans [22], 5 from Africans, and 11 from other races. In this analysis, Neanderthals were used as the outgroup (Fig 1). Our phylogenetic tree suggested that other races were separated from the African group, including modern-day African-Americans. Based on the differences in Glu12 frequency between the three groups, it is likely that most ancestors of modern-day Africans primarily had Glu12 repeats in the α2B-AR gene, and not the Glu9 repeats (Fig 1).

### Evolutionary research based on the α2B-AR: p.Glu 301–303 polymorphism in humans and primates

In recent years, the complete genomes of Neanderthals and the ancestors of humans were deciphered [23]. We analyzed the α2B-AR gene to determine its molecular evolution in chimpanzee and gorilla, that is, between closely related simian species. Moreover, we analyzed why two different Glu polymorphisms exist in modern humans and investigated the differences in the number of Glu repeats (8 and 10 repeats) in simian species (Table 3) [20, 24]. We also considered how bipedalism occurred and whether it is related to the development of NMS.

**Table 3:** Alignment of the α2B-AR sequences for the Glu repeat sites.

**Human: Glu12**																																													
**Homo sapiens; EU332847**	G	A	A	G	A	G	G	A	G	**G**	**A**	**A**	**G**	**A**	**G**	**G**	**A**	**G**	-	-	-	-	-	-	-	-	-	G	A	G	G	A	G	G	A	G	G	A	G	G	A	A	G	A	G
**Human: Glu9**																																													
**Homo sapiens; AF316895**	G	A	A	G	A	G	G	A	G	**G**	**A**	**G**	**G**	**A**	**G**	**G**	**A**	**G**	-	-	-	-	-	-	-	-	-	G	A	G	-	-	-	-	-	-	-	-	-	G	A	A	G	A	G
**Neanderthal: Glu9**																																													
**Homo sapiens neanderthalensis[25]**	G	A	A	G	A	G	G	A	G	**G**	**A**	**R**	**G**	**A**	**G**	**G**	**A**	**G**	-	-	-	-	-	-	-	-	-	G	A	G	-	-	-	-	-	-	-	-	-	G	A	A	G	A	G
**Chimpanzee: Glu10** ^**1**^																																													
**Pan troglodytes; GI:332813819**	G	A	A	G	A	G	G	A	G	**G**	**A**	**A**	**G**	**A**	**G**	**G**	**A**	**G**	-	-	-	-	-	-	-	-	-	G	A	G	G	A	G	-	-	-	-	-	-	G	A	A	G	A	G
**Gorilla: 6E+3V+6E**																																													
**Gorilla gorilla; GI:426336406**	G	A	A	G	A	G	G	A	G	**G**	**A**	**A**	**G**	**A**	**G**	**G**	**A**	**G**	G	T	G	G	T	G	G	T	G	G	A	G	G	A	G	G	A	G	G	A	G	G	A	A	G	A	A
**White gibbon: Glu8** ^**1**^																																													
**Hylobates lar; AM050851**	G	A	A	G	A	G	G	A	G	**G**	**A**	**G**	**G**	**A**	**G**	**G**	**A**	**G**	-	-	-	-	-	-	-	-	-	-	-	-	-	-	-	-	-	-	-	-	-	G	A	A	G	A	G
**Rhesus monkey: Glu8** ^**1**^																																													
**Macaca mulatta; AM050852**	G	A	A	G	A	G	G	A	G	**G**	**A**	**G**	**G**	**A**	**G**	**G**	**A**	**G**	-	-	-	-	-	-	-	-	-	-	-	-	-	-	-	-	-	-	-	-	-	G	A	A	G	A	G
**Codons to Amino Acids**	E:Glu			E			E			**E:301**			**E:302**			**E:303**			V:Val			V			V			E			E			E			E			E			E		

^1^ Amino acids number may vary species to species.

We found a Glu12/9 heterotype in the α2B-AR gene of 8 of the 9 total NMS subjects (S2 Table), inferring that this heterotype is related to the onset of NMS. However, it was not clear why two types of Glu repeats, Glu9 and Glu12, exist in modern humans. To clarify this problem, we examined the origin of these polymorphisms and when NMS began to occur in modern humans by obtaining the α2B-AR gene sequences of 4 simians in the NCBI and Ensembl databases: chimpanzee, white gibbon, rhesus monkey, and gorilla. We also obtained the α2B-AR gene sequence of Neanderthals from Ensembl’s “The Neanderthal Genome” database (chromosome:NCBI36:2:96144272:96145615:1) [25]. In addition, the sequences of 2 modern humans were included in the sequencing data. Next, these 7 sequences were aligned using CLUSTALW to confirm the number of Glu repeats (Table 3).

**Fig 2:**
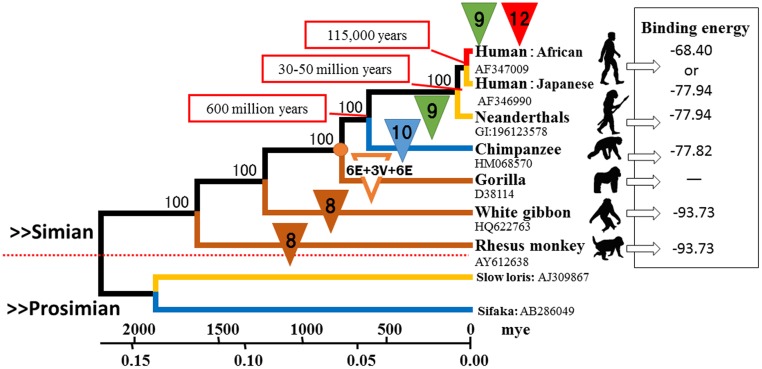
Phylogenetic tree constructed by the unweighted pair group method method showing the complete mtDNA region (16,750 bp) of 3 *Homo sapiens sapiens* and 6 primates and suggesting the evolution of the Glu9 and Glu12 repeats in the α2B-AR gene. The variability of the Glu repeats increased during the evolution of early primates to humans. This development likely changed the role of vasoconstriction in blood pressure while in an upright position.

First, we obtained 9 complete mitochondrial sequences including two prosimians from the databases and constructed a phylogenetic tree using the unweighted pair group method with arithmetic mean (UPGMA) method (Fig 2). This phylogenetic tree suggested the evolutionary genealogy of the simians examined and is in accordance with the results of other reports [21, 26–32].

The number of Glu repeats is displayed on the phylogenetic tree (Fig 2): 8 repeats in the white gibbon and rhesus monkey, 10 repeats in the chimpanzee, and 9 repeats in the Neanderthal.

**Fig 3:**
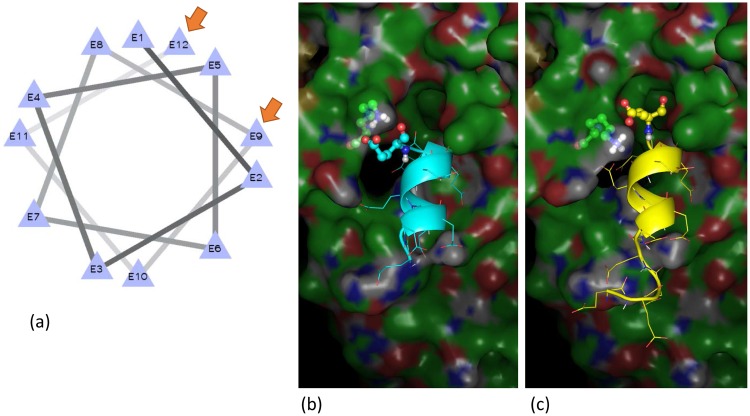
Helix wheel (a) and binding site of Glu9 (b) and Glu12 (c). The helix wheel is mostly unchanged functionally as Glu9 and Glu12 bind to the site in the adjoining position.

**Fig 4:**
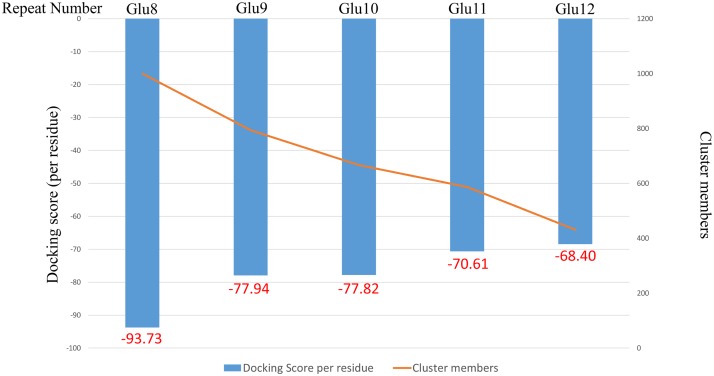
Binding energy of Gi α-subunit protein by *in silico* analysis. The receptor included in the Glu12 repeats can be released quickly from the β and γ subunits. The α subunit has a faster effect on adenylate cyclase signaling.

However, the gorilla sequence differed from that of the other simians as there were 3 valine (Val) repeats (GTG GTG GTG) between the Glu6 to Glu6 repeats area (Table 3). Therefore, Glu12 in the gorilla sample likely occurred by the insertion of 3 Val repeats and 4 Glu repeats when the gorilla species branched. Thereafter, the Glu12 repeats occurred in humans due to the deletion of the 3 Val residues during evolution. In addition, as shown in Figs 3 and 4, the binding energy of the Gi α-subunit protein (S1 Fig), as determined by *in silico* analysis, was -93.73 for Glu8, the same as that in the white gibbon and rhesus monkey, while it was approximately -77.82 for chimpanzee Glu10; it was -77.94 for Glu9 in humans. Human Glu12 resulted in the weakest bond of -68.40, resulting in the quickest dissociation rate from the Gi α-subunit (S1b Fig).

These results show that the dissociation value decreased as the number of repetitions increased and that the value indicates weak binding, causing AC to signal at a faster rate. Furthermore, the helix wheel (Fig 3a) is mostly unchanged functionally between Glu9 (Fig 3b) and Glu12 (Fig 3c), as shown by the location of the binding site in an adjoining position (S5 Fig). These results suggested our consideration of the Glu12 having an important role in sending smooth blood flows to the brain. The need for smooth blood flows to the brain arose from changes in the species gradual ascension of their head position.

According to our results, Glu8 repeats existed until the gorilla branch node, after which the Glu12 repeats may have occurred over time by repeating the deletion and duplication in the course of evolution (Fig 2). However, since the number of Neanderthal sample that was analyzed was small, we could only estimate that Glu12 evolved when early hominids, the ancestors of Neanderthals and modern humans, split from the rest of the primate lineage approximately 30−50 million years ago [23, 33, 34]. In addition, our analysis revealed that ancient Africans had already developed Glu12 repeats in α2B-AR. According to the results of Nei and Roychoudhury [35], other human populations diverged from Africans 115,000 years ago, suggesting that NMS existed in ancient Africans as early as 115,000 years ago.

### Analysis of protein structures in the α2B-AR gene and Gi α-subunit and subunit binding energy

We elucidated the mechanism of molecular interactions based on the polymorphisms of the α2B-AR gene and Gi protein in NMS. The Gi protein serves an important role in the activity of AC. Mainly, Gi inhibits the cAMP-dependent pathway by inhibiting the activity of AC and decreasing cAMP production from ATP, which also results in decreased cAMP-dependent protein kinase activity [15, 36, 37]. Therefore, the decrease in calcium concentration is connected to the decrease in the contractile force of the smooth muscle (S1 Fig).

Accordingly, we analyzed secondary structure and performed disorder prediction to investigate the characteristics of the protein structure of the intracellular third loop containing the Glu repeats (S5a Fig). From the results of DISOPRED2, the intracellular third loop was predicted to have a disordered structure (S5b and S5c Fig).

Conversely, the secondary structure of the poly-Glu repeats-specific area was predicted to be helical. Consequently, only the Glu repeats region was predicted to form a local helical structure (S6 Fig), and the results of helix wheel analysis indicated that the helix wheel is unchanged functionally between Glu9 and Glu12, as shown by the location of the binding site in the same position (Fig 3). Therefore, the existence of the different genotypes has caused no functional problems in humans.

From the G protein-coupled receptor (GPCR) and co-crystal structure of the G protein, the intracellular third loop is expected to interact with the surfaces of the G protein exposed to the cytoplasm. Therefore, we predicted the interaction energy and point of attachment of the Glu repeats to the G protein by conducting docking simulations. ClusPro revealed that both Glu12 and Glu9 repeats bound to a specific position, namely, the Gi α-subunit (S5b and S5c Fig). We also clarified that the binding energy per residue was approximately -68.40 in Glu12 and -77.94 in Glu9 [38]. The binding energy of the Gi α-subunit in Glu9 was stronger than in Glu12 (Fig 4). These results suggest that the receptor included in the Glu12 repeats can be released quickly from the β and γ subunits and that the α subunit has a faster effect on AC signaling (S1b Fig).

During the HUT test, the number of Glu12 repeats increased as orthostatic blood pressure dropped and heart rate increased (S3 Table). It seems that subjects with the Glu9/9 homotype have a greater degree of heart rate increase than those with the Glu12 repeats. The differences in heart rate were higher in Glu9/9 than in Glu12/12 and Glu12/9 (25 ± 15 bpm, 6 ± 19 bpm, and 14 ± 10 bpm, respectively). However, as the number of Glu9/9 subjects (3 controls) was small, we were unable to show a significant difference (p value = 0.259). Consequently, we failed to reject the null hypothesis at the 5% level (S3 Table).

Nevertheless, our data are in agreement with those of Tayel et al. [39], who conducted a study to determine the association between the hetero polymorphism of the α2B-AR gene and hypertension in patients with and without diabetes. They assessed only one marked and significant association between the Glu9/9 genotype and Glu12/9 polymorphisms in the α2B-AR gene, which may be a possible risk factor for hypertension with and without diabetes. In other words, they suggested that subjects with the Glu9/9 homo genotype had a higher risk of hypertension. We suggest that, in the presence of both genotypes, a deviation occurs at the time of the dissociation of α subunit, and suppression time, which increases adenylyl cyclase activity, is longer than in individuals with the homo genotypes (S1 Fig).

## Discussion

The purpose of this study was to investigate whether NMS is caused by acute stress. Our results showed that subjects with Glu12 repeats are more vulnerable than subjects with Glu9 repeats to orthostatic stress. In particular, we obtained interesting results from our clinical research; 8 of the 9 total NMS subjects had the Glu12/9 heterotype [4].

In general, sympathetic activity occurs in blood vessels thinned by contracting smooth muscle and contraction of blood vessels. However, NMS occurs when agitated sympathetic activity extends blood flow and inhibits blood vessel contraction. Therefore, during stress, Ad and NA affect vascular contraction and relaxation of smooth muscle by means of the respective α2B-AR gene receptors in the Glu12/9 heterotype and Glu9/9 and Glu12/12 homotypes. Several studies have reported that when NA is administered, AC activity is not suppressed in individuals with the Glu9/9 homotype, but is suppressed in those with Glu12/12 [20, 40].

According to our hypothesis, we considered that NMS is relieved by the passage of time. However, in the case of the Glu12/9 heterotype, there are two different receptor types. We then compared the binding energy of the α2B-AR gene receptors between Glu12 and Glu9.

Protein analysis revealed that the binding energy of each residue was approximately -68.40 for Glu12 and -78.94 for Glu9. The binding energy of the Gi α-subunit with Glu9 was higher than with Glu12, suggesting that the receptor with Glu12 can be released quickly from the Gi α-subunit. The binding energy of the Gi α-subunit has decreased from rhesus monkey (-93.73) to humans (-68.40) during evolution. In other words, the α-subunit has a faster effect on AC signaling.

Our data suggest that the Glu12/9 heterotype suppressed the action of AC longer than the homotype, which we determined by measuring the differences in the dissociation time of the Gi α-subunit (S1 Fig). This effect is thought to be one of the causes of NMS. In regard to the acquisition of bipedalism in humans, the high position of the head requires higher blood pressure to maintain an adequate blood supply (Fig 2). As humans need to increase blood flow to dilate the blood vessels, the Glu12 receptor type has become necessary to suppress vasoconstriction.

The results also suggest that Glu8 was originally repeated through the gorilla branch node and that the Glu12 repeats occurred over time by repeating the deletion and duplication during evolution (Table 3 and Fig 2). Via the phylogenetic tree, we presumed that the frequency of the Glu12 repeats of the α2B-AR gene was high in the ancestors of Africans, compared with Glu9 (S1 Table). Therefore, we surmised that the Glu12 repeats of the α2B-AR gene have existed since the first humans. On the basis of gene frequency and the phylogenetic tree of humans, ancient Africans had developed the Glu12 repeats of α2B-AR as early as 115,000 years ago. In addition, the use of vasoconstriction as a means of regulating blood pressure likely developed as a result of acquiring the 12 repeats and developing bipedalism.

As humans can be overcome by dizziness when we stand up suddenly, we speculate that this dizziness is necessary to maintain balance so as to prevent falling by regulating blood pressure. In other words, our tentative theory is that syncope is a product of evolutionary development and should not be considered a disease, and it is thus a passive defense mechanism. In addition, the present findings will be useful for contributing to the standardization of medical management regarding syncope during large-scale disasters in Japan, which are anticipated in the future.

## Conclusion

Subjects with NMS who have Glu12 repeats could be more vulnerable to orthostatic stress than subjects who have Glu9 repeats. In addition, in the NMS subjects, there were 8 with the Glu12/9 heterotype and 1 with the Glu12/12 homotype, while the Glu9/9 genotype was not observed. Moreover, this study also suggested that NMS was affected by the suppression of AC signaling, which increases from the difference in the two rates of dissociation of Gi (α) for the Glu12/9 heterotype. Also, according to the gene frequency ratio and phylogenetic tree, we hypothesized that many ancestors of Africans had a high frequency of the Glu12 repeats of α2B-AR. Moreover, the number of ancestors with the Glu9 polymorphism was originally low. Therefore, we surmised that the Glu12 repeats of α2B-AR were originally found in humans. As a consequence, we considered that NMS is produced as a defensive measure caused by the acquisition of α2B-AR Glu12 repeats preventing overly-significant blood flow to the human brain during the evolution of the bipedal upright position. This defensive measure had already developed by 115,000 years ago.

## Materials and Methods

### Ethics statement

Our studies were approved by the respective institutional Ethics Committees. All subjects gave written consent including for clinical researches and genetic studies.

This study was reviewed and approved by the research ethics board of each university.

All 20 subjects were collected from mainland Japan and provided informed consent and these healthy volunteers provided informed consent prior to sampling, following approval of the experimental procedure by the relevant ethics committee at Tokai University.

### Clinical research of NMS subjects and controls

#### Subjects

Nine ambulatory patients with NMS were recruited from the Department of Medicine, Tokyo Women’s Medical University in accordance with the guidelines on the management of NMS [41] and were compared with 11 healthy subjects recruited from the Tokai University School of Medicine and the Department of Medicine, Tokyo Women’s Medical University (S2 Table). All 20 subjects were collected from mainland Japan and provided written informed consent.

This study was reviewed and approved by the research ethics board of each university. All study participants fulfilled the following criteria: 1) history of ≥2 episodes of syncope or presyncope in the upright position within the last 6 months; 2) no evidence of structural cardiovascular disease by patient history, physical examination, echocardiography, and 24-h Holter electrocardiography; and 3) unremarkable work-ups for other known causes of syncope, including laboratory tests and neurological examinations.

#### General protocol

The experiments were carried out at Tokyo Women’s Medical University Medical Center East and the Tokai University School of Medicine. All medications that might have affected the autonomic nervous system were withheld for at least 5 administrations to ensure sufficient elimination before the experiments. Standardized cardiovascular autonomic function tests were performed as described previously [42]. Continuous blood pressure and heart rate measurements were obtained using an automated oscillometric sphygmomanometer and tonometry (BP-608 Evolution II; Omron Colin Corporation, Tokyo, Japan).

A tilt table was inclined in 10-min stages: 0° (baseline), 20°, 40°, and 60°. Plasma Ad and NA concentrations were measured in the supine position after 30 min and at the end of the HUT test by an external research institution. All tests were performed over a 3-h period for each subject (S2 Fig) [4].

#### Statistical analysis

Descriptive analyses of baseline characteristics were performed for each continuous variable using Wilcoxon’s signed-rank sum test. The chi-squared test was performed for nominal variables. Values are reported as the means ± standard deviation unless noted otherwise. P values < 0.05 were considered significant, and all tests were two-tailed. Statistical analysis was performed using JMP for Windows version 11 (SAS Institute Inc., Cary, NC).

### Molecular biological analysis

#### DNA extraction, PCR amplification, sequencing, and cloning

We evaluated the genetic polymorphisms of the α2B-AR gene as well as cardiovascular autonomic function in 11 control subjects who had never experienced syncope and 9 NMS patients referred to our outpatient department. A blood sample was taken from each of the 20 subjects and suspended in 400 μl TNES-8 M urea. Each sample was subsequently mixed with 500 μl phenol/chloroform/isoamyl alcohol (25:24:1) for 3 min, and the procedure was repeated twice. After precipitation with 2–2.5 v/v ethanol, the pellets were rinsed in 70% cold ethanol and dried. The samples were then dissolved in TE buffer (10 mM Tris-HCl pH 8.0, 1 mM EDTA).

Two sets of primers (α2B-AR_1F, 5′-GGGCGACGCTCTTGTCTA-3′ [forward] and α2B-AR_1R, 5′-ACTTCGAGTGTCCGTTGACC-3′ [reverse]; and α2B-AR_2F, 5′-ATCTACCTG ATCGCCAAACG-3′ [forward] and α2B-AR_2R, 5′-ATGAGGCCTACAGGATCTGG-3′ [reverse]) were designed to amplify a fragment of the α2B-AR gene on the basis of the human adrenergic α2B-AR receptor gene sequence from GenBank at the National Institutes of Health (Bethesda, MD). A Takara Ex Taq Reaction Kit (Takara Bio, Shiga, Japan) was used for polymerase chain reaction (PCR) enzymes, and PCR was performed under the following conditions: denaturation at 95°C for 3 min followed by 35 cycles of 10 s at 0095°C, annealing at 64°C for 20 s, and extension at 72°C for 30 s. A PCR product of the expected size (1,571 bp) was amplified with the primers. The band corresponding to the PCR product was excised from a 1.0% agarose gel (Nacalai Tesque, Kyoto, Japan). After purification with MACHEREY-NAGEL NucleoSpin Extract II (Düren, Germany), the products were sequenced directly using Big Dye Terminator Ver. 3.1 and an ABI Prism 3500xl DNA sequencer (ABI, Foster City, CA).

#### Microsatellite genotyping and DNA samples

We analyzed 281 DNA samples from the Japanese Collection of Research Bioresources (Osaka, Japan) for microsatellite genotyping. The samples were mapped by the following method. The forward primer (5′-TGAGGCCGGAGACACTGGC-3′) was used to amplify the microsatellites and labeled with 5′-fluorescent FAM, while the reverse primer (5′-CCAGTTGGGCTGCCCTTCC-3′) was not labeled. The PCR reaction used 4 ng genomic DNA, 20 pmol forward/reverse primers, 1 μl dNTP (2.5 mM each), 1 μl 10× buffer, and 0.5 U AmpliTaq Gold DNA polymerase (Life Technologies, Tokyo, Japan) in a total volume of 10 μl. After initial denaturation for 9 min at 95°C, amplification was carried out in an automated thermal cycler (Life Technologies) for 40 cycles of 30 s at 96°C, 30 s at 66°C, and 30 s at 72°C, with a final extension of 5 min at 72°C. Fragment analysis by capillary electrophoresis using a 3730 Genetic Analyzer (Life Technologies) and allele assignment using GeneMapper Software (Life Technologies) have been described previously.

As fragment size was estimated, we confirmed the alleles by using direct PCR sequencing. Two fragments selected in each homozygote estimated by microsatellite genotyping were sequenced with standard direct PCR sequencing (S4 Fig).

#### Alignment of α2B-AR gene sequences and complete mtDNA

The 20 nucleotide sequences of α2B-AR gene, 1 from each of the aforementioned 20 subjects, were aligned using CLUSTALW [43]. Common sites in all sequences where gaps were found were excluded from analysis [20, 23–25, 44, 45]. The aligned sites of each sequence comprised approximately 1,000 bp (Table 3). Next, the number of nucleotide substitutions between each pair of aligned sequences was computed using Kimura’s two-parameter method [46]. These complete mtDNA sequences (16,750 bp) (Figs 1 and 2) [21, 47] were multiple aligned using CLUSTALW [43].

#### Molecular phylogeny analysis of complete mtDNA

A phylogenetic tree (Fig 1) was constructed by the neighbor-joining (NJ) method [48], and the NJ algorithms were incorporated into the MEGA6 package using the distances corrected for multiple hits based on Kimura’s two-parameter model [46, 49]. Sites representing a gap in any of the aligned sequences were excluded from the analysis.

A second phylogenetic tree (Fig 2) was constructed by the UPGMA method, and the UPGMA algorithms were incorporated into the MEGA6 package using the distances corrected for multiple hits based on Kimura’s two-parameter model [46, 49]. Both phylogenetic trees used bootstrap analysis of 1,000 replications to assess statistical confidence in the branching order of the trees.

#### Analysis of protein structure

We predicted secondary structure and disorder to investigate the characteristics of the protein structure of the intracellular third loop containing the Glu repeats. For the analysis of disorder prediction, we used DISOPRED2 [50], and for the analysis of the secondary structure, we predicted protein structure using PSI-PRED [51].

As we hypothesized that the Glu repeats forms a helix structure, we prepared a helical structure of the 8–12 residues in each poly-Glu repeats sequence by artificial measures. The three-dimensional structures of the amino acid variants from the 8–12 poly-Glu repeats were constructed using the MOE program with default parameters (Chemical Computing Group, Montreal, Canada). The structural models of the 8–12 poly-Glu repeats were docked into the X-ray structure of the Gi α-subunit from the co-crystal structure of the GPCR and G proteins (PDB-ID: 3sn6) [52] using the docking simulation program ClusPro (Boston University, Boston, MA) [53] with the “Electrostatic Favored” interaction energy score.

## Limitations

Our data were derived from a small number of NMS patients. We performed biochemical and HUT tests in each syncope subject in a single day to determine which of them had NMS. It was necessary to proceed with caution during the HUT test because it is risky in such patients. The symptoms of NMS occur during a stressful situation. We would like for you to understand beforehand that there is a limit to the number of samples used in this experiment [4]. Healthy subjects with poor physical performance in the HUT test were excluded from data analysis

## Supporting Information

S1 Fig Mechanism of activation and inhibition of cAMP by the Gi and Gs subunits. (a) Effect of the polymorphism of the α2B-AR gene on neurally mediated syncope. (b) Relationship between vasoconstriction and adenylate cyclase (AC) activity. Adenylate cyclase (AC) mediates the effects of Gi protein on the contraction and relaxation of blood vessels by acting on adrenergic receptor α1 or β2. When the adrenergic subtype β2 receptor is activated, it activates the binding of AC to Gs protein to create cyclic adenosine monophosphate (cAMP).
(TIF)

S2 Fig Schematic illustration of the graded head-up tilt (HUT) test. The tilt table was inclined in 10-min stages: 0° (baseline), 20°, 40°, and 60°. Plasma adrenaline and noradrenaline were measured in the supine position after 30 min and at the end of the HUT test.
(TIF)

S3 Fig Plasma noradrenaline (NA) and adrenaline (Ad) concentrations before and after the HUT test. (a) Plasma Ad concentration before and after the head-up tilt (HUT) test in the NMS subjects and controls. (b) Plasma NA concentration before and after the HUT test in the NMS subjects and controls. (c) Plasma Ad concentration before and after the HUT test in the subjects with Glu12/12, Glu12/9, and Glu9/9 genotypes. (d) Plasma NA concentration before and after the HUT test in the subjects with Glu12/12, Glu12/9, and Glu9/9 genotypes. The NMS patients showed high Ad and NA concentrations before and after the HUT test. Subjects with Glu12 repeats had higher NA concentrations than those with Glu9 repeats among the three different genotypes in humans (Glu12/12, Glu12/9, and Glu9/9).
(TIF)

S4 Fig Identification of the α2B-AR: p.Glu 301–303 polymorphism by automated DNA sequencing. (a) Polymorphism of the Glu9 repeats. (b) Polymorphism of the Glu12 repeats. We confirmed the alleles by using direct polymerase chain reaction (PCR) sequencing. Two fragments selected in each homozygote estimated by microsatellite genotyping were sequenced with standard direct PCR sequencing.
(TIF)

S5 Fig Secondary structure (a) and binding site of Glu9 (b) and Glu12 (c). Secondary structure (a) and disorder prediction of the protein structure of the intracellular third loop containing the Glu9 (b) and Glu12 (c) repeats.
(TIF)

S6 Fig Simplified illustration of the prediction of the secondary structure around the repeat region. The secondary structure of the poly-Glu repeats-specific area was predicted to be helical. Consequently, only the Glu repeats region was predicted to form a local helical structure.
(TIF)

S1 Table Frequency of the α2B-AR: del 301–303 allele frequency among three populations using the Caucasian and African-American data of Small’s study [20]. ^1^ Data from our experiment pertains to the Japanese population.
(DOCX)

S2 Table Subject characteristics. (DOCX)

S3 Table Blood pressure and heart rate before and after the HUT test. ^1^A is before the HUT test. B is the difference after the HUT test. ^2^SBP: systolic blood pressure; ^3^DBP: diastolic blood pressure; ^4^HR: heart rate; HUT, head-up tilt
(DOCX)
