# Supplementary figures and images for: Relationship between Human Evolution and Neurally Mediated Syncope Disclosed by the Polymorphic Sites of the Adrenergic Receptor Gene α2B-AR
Source: PLoS One. 2015 Apr 10;10(4):e0120788. doi: 10.1371/journal.pone.0120788 (PMC4393242; doi:10.1371/journal.pone.0120788)

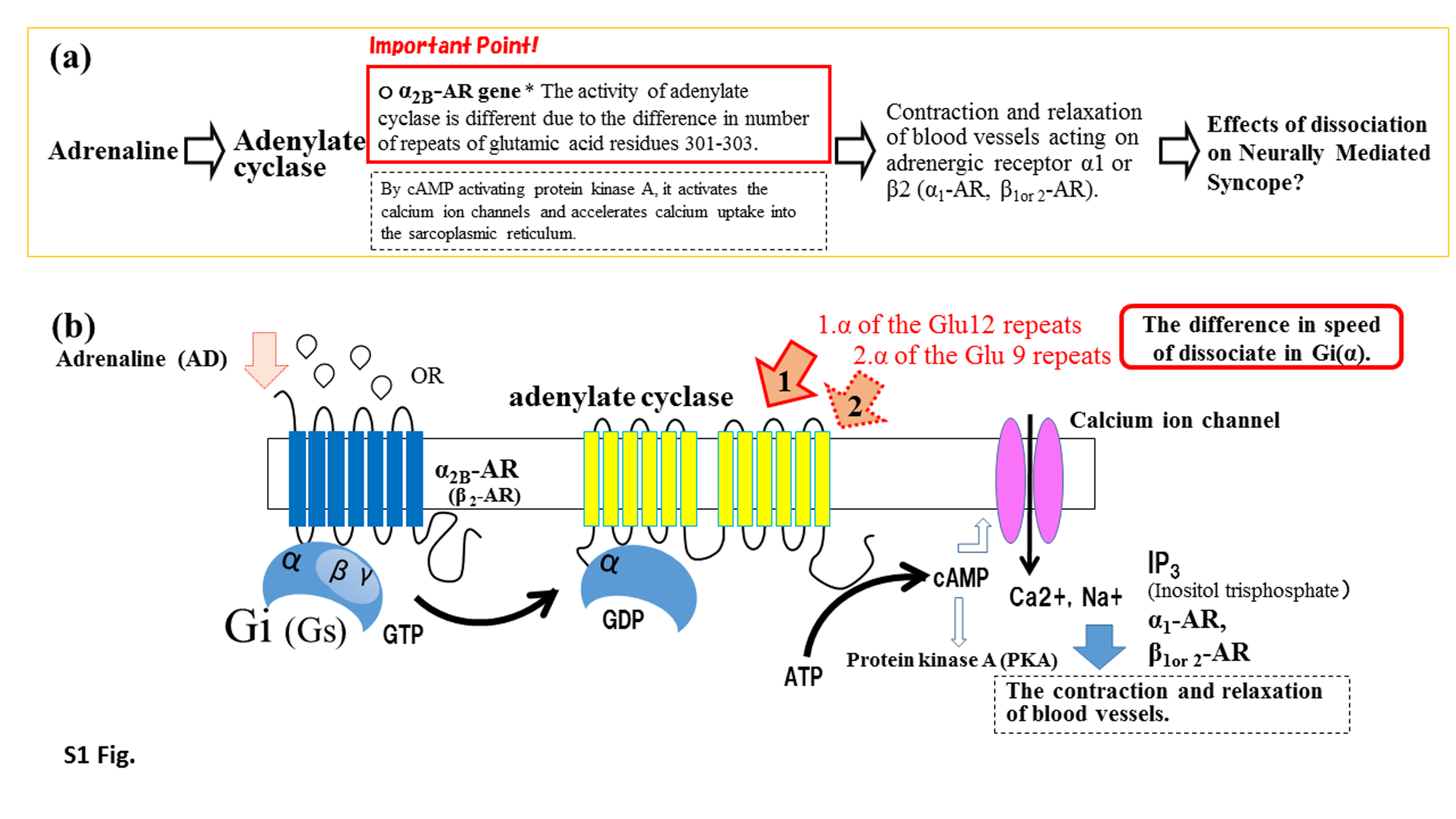

Supplement: S1 Fig — (a) Effect of the polymorphism of the α2B-AR gene on neurally mediated syncope. (b) Relationship between vasoconstriction and adenylate cyclase (AC) activity. Adenylate cyclase (AC) mediates the effects of Gi protein on the contraction and relaxation of blood vessels by acting on adrenergic receptor α1 or β2. When the adrenergic subtype β2 receptor is activated, it activates the binding of AC to Gs protein to create cyclic adenosine monophosphate (cAMP). (TIF) [file pone.0120788.s001.tif]

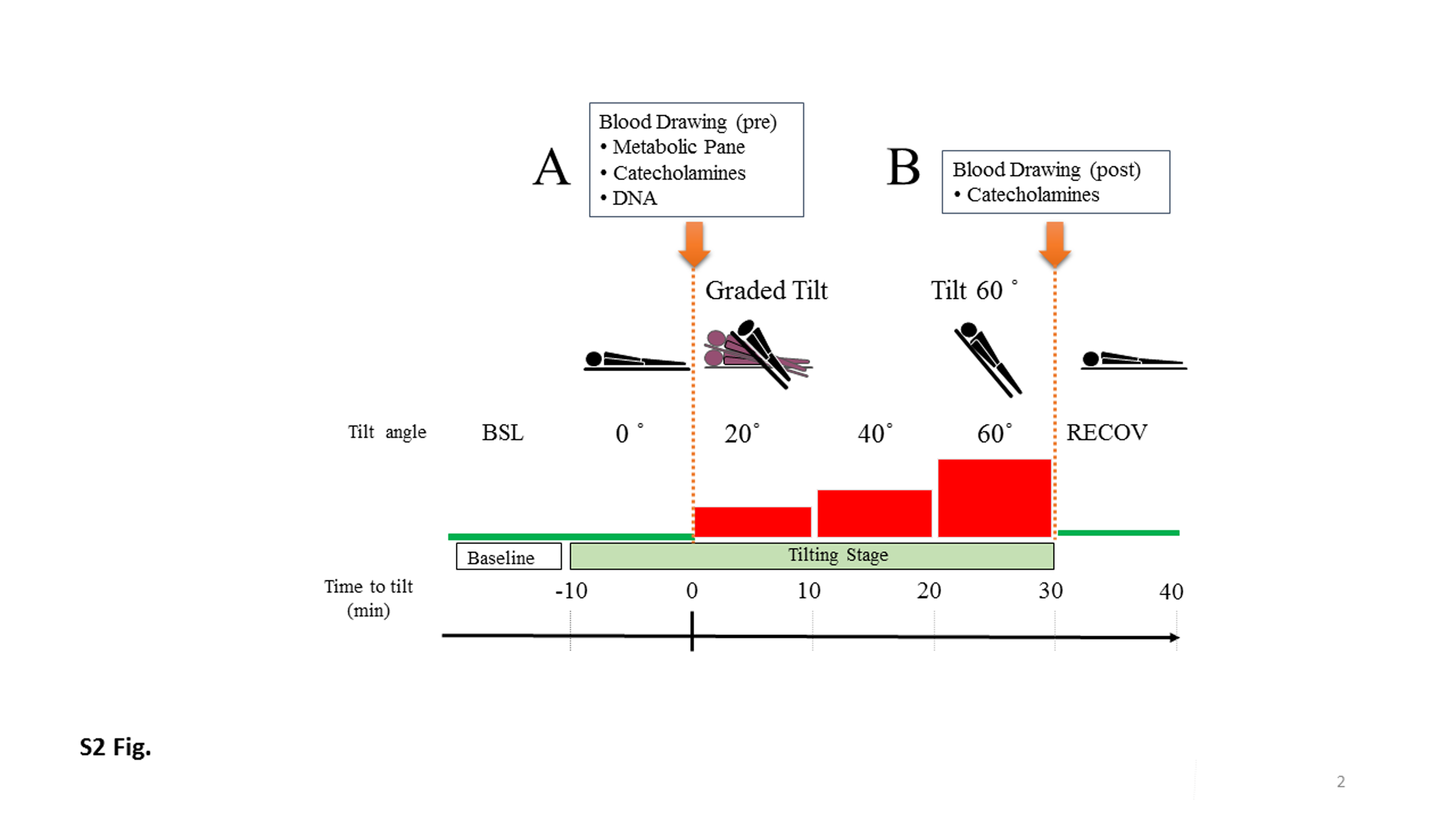

Supplement: S2 Fig — The tilt table was inclined in 10-min stages: 0° (baseline), 20°, 40°, and 60°. Plasma adrenaline and noradrenaline were measured in the supine position after 30 min and at the end of the HUT test. (TIF) [file pone.0120788.s002.tif]

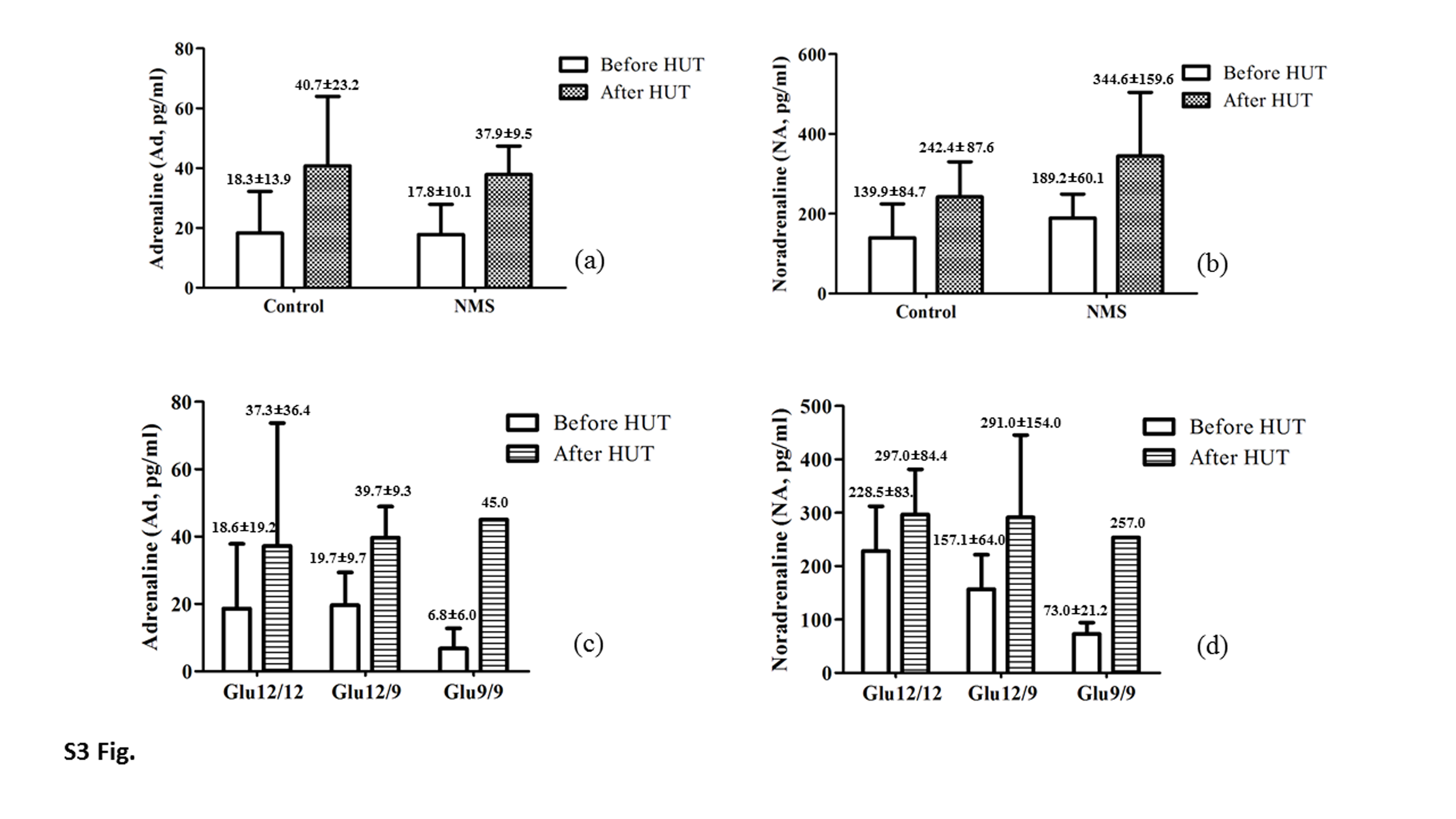

Supplement: S3 Fig — (a) Plasma Ad concentration before and after the head-up tilt (HUT) test in the NMS subjects and controls. (b) Plasma NA concentration before and after the HUT test in the NMS subjects and controls. (c) Plasma Ad concentration before and after the HUT test in the subjects with Glu12/12, Glu12/9, and Glu9/9 genotypes. (d) Plasma NA concentration before and after the HUT test in the subjects with Glu12/12, Glu12/9, and Glu9/9 genotypes. The NMS patients showed high Ad and NA concentrations before and after the HUT test. Subjects with Glu12 repeats had higher NA concentrations than those with Glu9 repeats among the three different genotypes in humans (Glu12/12, Glu12/9, and Glu9/9). (TIF) [file pone.0120788.s003.tif]

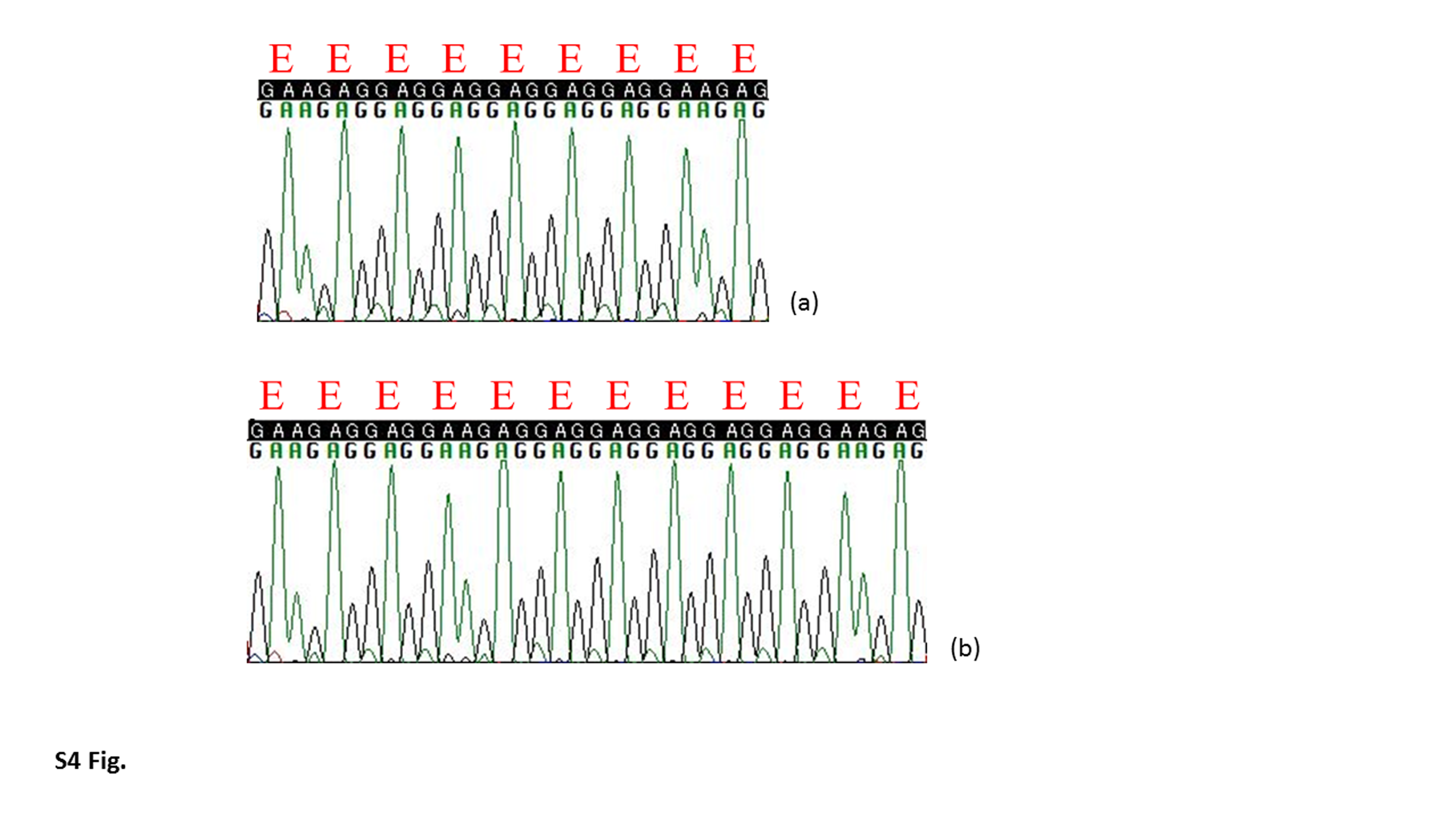

Supplement: S4 Fig — (a) Polymorphism of the Glu9 repeats. (b) Polymorphism of the Glu12 repeats. We confirmed the alleles by using direct polymerase chain reaction (PCR) sequencing. Two fragments selected in each homozygote estimated by microsatellite genotyping were sequenced with standard direct PCR sequencing. (TIF) [file pone.0120788.s004.tif]

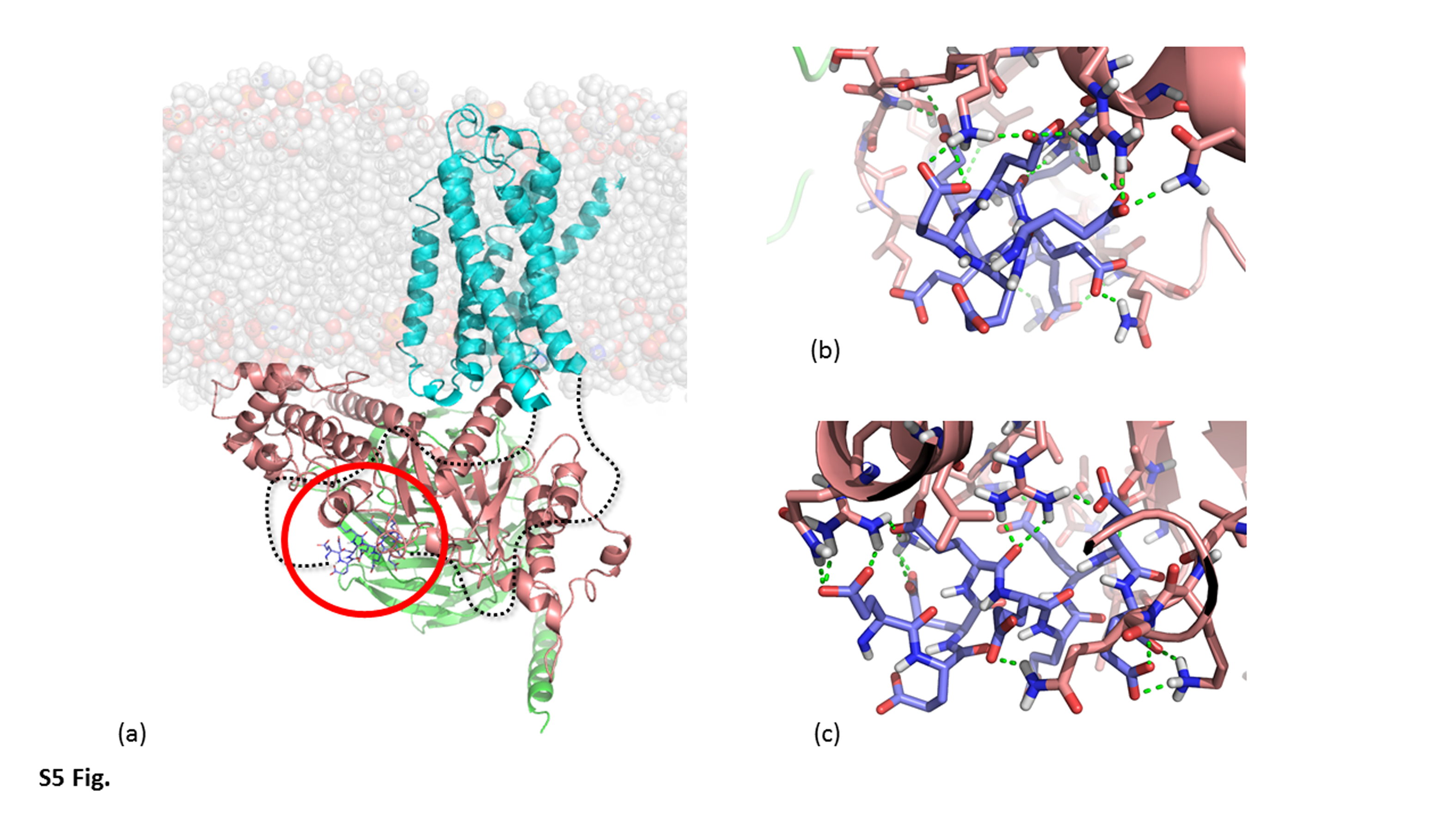

Supplement: S5 Fig — Secondary structure (a) and disorder prediction of the protein structure of the intracellular third loop containing the Glu9 (b) and Glu12 (c) repeats. (TIF) [file pone.0120788.s005.tif]

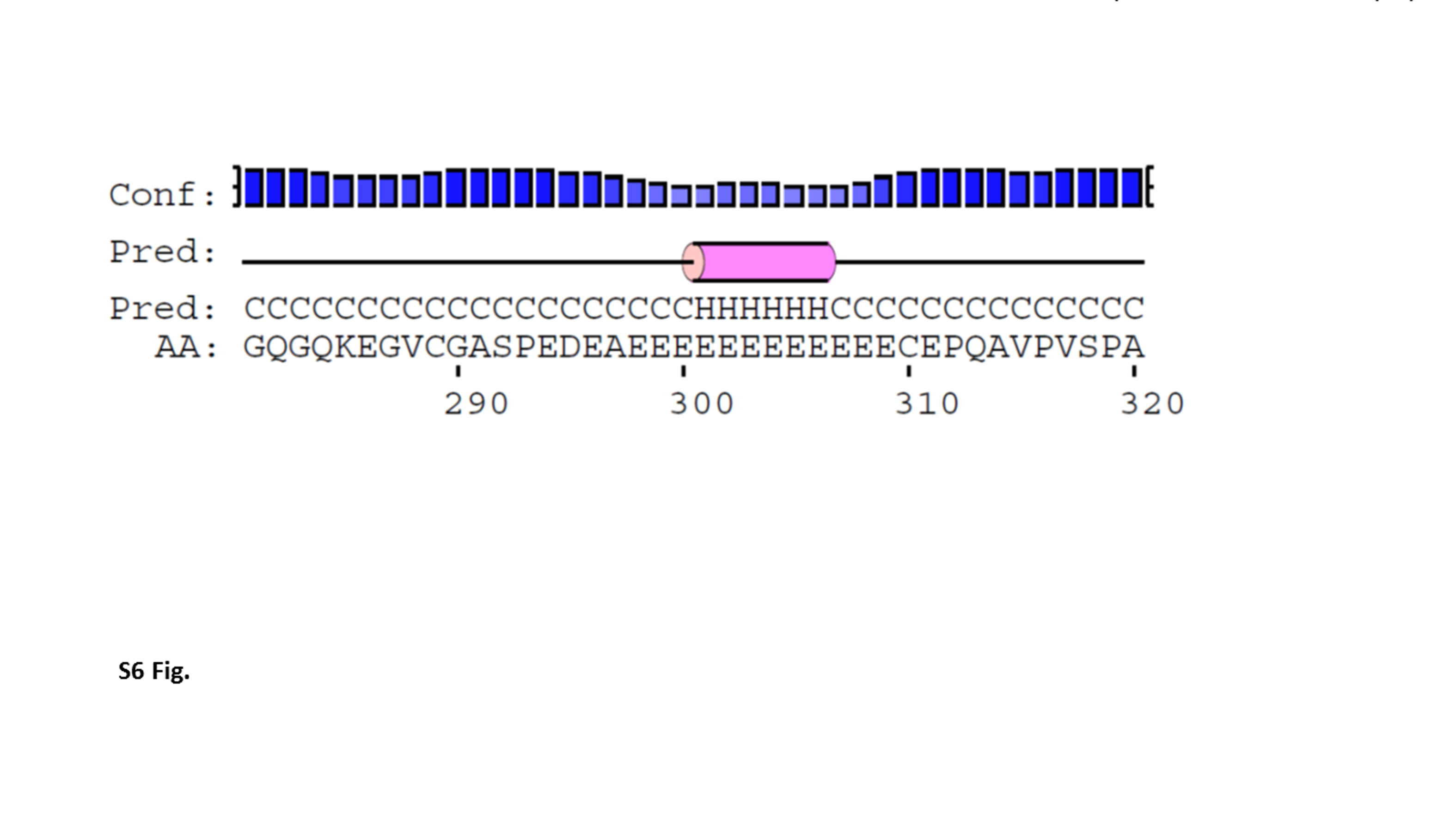

Supplement: S6 Fig — The secondary structure of the poly-Glu repeats-specific area was predicted to be helical. Consequently, only the Glu repeats region was predicted to form a local helical structure. (TIF) [file pone.0120788.s006.tif]
