# Supplementary material for: Relationship between Human Evolution and Neurally Mediated Syncope Disclosed by the Polymorphic Sites of the Adrenergic Receptor Gene α2B-AR
Source: PLoS One. 2015 Apr 10;10(4):e0120788. doi: 10.1371/journal.pone.0120788 (PMC4393242; doi:10.1371/journal.pone.0120788)
Supplement: S1 Table — 1 Data from our experiment pertains to the Japanese population. (DOCX) [file pone.0120788.s007.docx]

| **S1 Table.** Frequency of the α2B-AR: del 301–303 allele frequency among three populations using the Caucasian and African-American data of Small’s study (20). | | | | | |
| --- | --- | --- | --- | --- | --- |
| **Population Name** | ***n*** | **Homozygous 12/12** | **Heterozygous 12/9** | **Del 301–303 homozygous 9/9** | **Del 301–303 allele frequency** |
| Caucasian | 94 | 41 | 47 | 6 | 0.31 |
| African-American | 79 | 61 | 17 | 1 | 0.12 |
| Japanese^1^ | 281 | 109 | 127 | 45 | 0.39 |

^1^Japanese are our data.
