# Supplementary material for: Relationship between Human Evolution and Neurally Mediated Syncope Disclosed by the Polymorphic Sites of the Adrenergic Receptor Gene α2B-AR
Source: PLoS One. 2015 Apr 10;10(4):e0120788. doi: 10.1371/journal.pone.0120788 (PMC4393242; doi:10.1371/journal.pone.0120788)
Supplement: S2 Table — (DOCX) [file pone.0120788.s008.docx]

| **S2 Table.** Subject characteristics. | | | | | | |
| --- | --- | --- | --- | --- | --- | --- |
|  | **n** | | **Age (years)** | **Glu12/12** | **Glu12/9** | **Glu9/9** |
| Control | 11 | Male 7 | 29.0±4.4 | 1 | 5 | 1 |
|  |  | Female 4 | 38.0±10.6 | 2 | 0 | 2 |
| NMS | 9 | Male 5 | 44.6±13.0 | 0 | 5 | 0 |
|  |  | Female 4 | 31.5±2.7 | 1 | 3 | 0 |
