# Supplementary material for: Relationship between Human Evolution and Neurally Mediated Syncope Disclosed by the Polymorphic Sites of the Adrenergic Receptor Gene α2B-AR
Source: PLoS One. 2015 Apr 10;10(4):e0120788. doi: 10.1371/journal.pone.0120788 (PMC4393242; doi:10.1371/journal.pone.0120788)
Supplement: S3 Table — 1A is before the HUT test. B is the difference after the HUT test. 2SBP: systolic blood pressure; 3DBP: diastolic blood pressure; 4HR: heart rate; HUT, head-up tilt (DOCX) [file pone.0120788.s009.docx]

| **S3 Table.** Blood pressure and heart rate before and after the HUT test. | | | | | |
| --- | --- | --- | --- | --- | --- |
|  | Repeat number | Glu12/12 | Glu12/9 | Glu9/9 | p-value |
|  | n | 4 | 13 | 3 |  |
| A^1^ | Baseline SBP^2^ (mmHg) | 116±16 | 120±11 | 114±6 | 0.610 |
|  | Baseline DBP^3^ (mmHg) | 65±11 | 71±13 | 62±6 | 0.241 |
|  | Baseline HR^4^(bpm) | 59±5 | 62±8 | 61±8 | 0.751 |
| B^1^ | dSBP^2^ (mmHg) | -18±21 | -9±24 | -8±19 | 0.437 |
|  | dDBP^3^ (mmHg) | -6±10 | -3±11 | 10±14 | 0.307 |
|  | dHR^4^(bpm) | 6±19 | 14±10 | 25±15 | 0.259 |
| ^1^A is before the HUT test. B is the difference after the HUT test. ^2^SBP: systolic blood pressure; ^3^DBP: diastolic blood pressure; ^4^HR: heart rate; HUT, head-up tilt | | | | | |
|  |  |  |  |  |  |
